# Mean Affect Moderates the Association between Affect Variability and Mental Health

**DOI:** 10.1007/s42761-024-00238-0

**Published:** 2024-06-13

**Authors:** Brooke N. Jenkins, Lydia Q. Ong, Anthony D. Ong, Hee Youn (Helen) Lee, Julia K. Boehm

**Affiliations:** 1 Department of Psychology, Crean College of Health and Behavioral Sciences, Chapman University, One University Drive, Orange, CA USA; 2 Center on Stress & Health, University of California, Irvine, CA USA; 3 Department of Anesthesiology and Perioperative Care, University of California, Irvine, Orange, CA USA; 4 Department of Psychology, University of British Columbia, Vancouver, BC Canada; 5 Department of Psychology, Cornell University, Ithaca, NY USA

**Keywords:** Affect, Affect variability, Mental health, Affect dynamics

## Abstract

Increasing evidence suggests that within-person variation in affect is a dimension distinct from mean levels along which individuals can be characterized. This study investigated affect variability’s association with concurrent and longitudinal mental health and how mean affect levels moderate these associations. The mental health outcomes of depression, panic disorder, self-rated mental health, and mental health professional visits from the second and third waves of the Midlife in the United States Study were used for cross-sectional (*n* = 1,676) and longitudinal outcomes (*n* = 1,271), respectively. These participants took part in the National Study of Daily Experiences (NSDE II), where they self-reported their affect once a day for 8 days, and this was used to compute affect mean and variability. Greater positive affect variability cross-sectionally predicted a higher likelihood of depression, panic disorder, mental health professional use, and poorer self-rated mental health. Greater negative affect variability predicted higher panic disorder probability. Longitudinally, elevated positive and negative affect variability predicted higher depression likelihood and worse self-rated mental health over time, while greater positive affect variability also predicted increased panic disorder probability. Additionally, mean affect moderated associations between variability and health such that variability-mental health associations primarily took place when mean positive affect was high (for concurrent mental health professional use and longitudinal depression) and when mean negative affect was low (for concurrent depression, panic disorder, self-rated mental health, and longitudinal self-rated mental health). Taken together, affect variability may have implications for both short- and long-term health and mean levels should be considered.

**Supplementary Information:**

The online version contains supplementary material available at 10.1007/s42761-024-00238-0.

Positive and negative affect are inherently dynamic, fluctuating over time (Ebner-Priemer et al., 2009; Pressman et al., 2017). These temporal shifts can be quantified as affect variability using metrics like the standard deviation of affect levels across multiple timepoints (Röcke et al., 2009). Considerable research shows greater variability in positive and negative affect predicts poorer mental health outcomes (Gruber et al., 2013; Houben et al., 2015; Jenkins et al., 2020; Koval, Pe, Meers, & Kuppens 2013; Kuppens et al., 2007; Peeters et al., 2006; Reitsema et al., 2022; Wichers et al., 2010). Such findings support the Stability Theory proposing variability reflects poor emotion regulation that may impair health (Gruber et al., 2013; Hardy & Segerstrom, 2017; Houben et al., 2015). However, the bulk of this research has focused on the main effect of affect variability on depression. This investigation tests the moderating role of mean affect levels, as well as curvilinear associations between affect variability and mental health using cross-sectional and longitudinal data. We also extend the literature by using three additional mental health outcomes: panic disorder (another pertinent mental health outcome), the holistic assessment of self-rated overall mental health, and mental health professional visits (which give insight into a person’s mental health status given that poor mental health is linked to greater uses of mental healthcare services (Wang et al., 2000)).

While the Stability Theory of Affect proposes a main effect of affect variability on health, the Fragile Desirable Affect Theory proposes that mean affect may moderate this link (Jenkins et al., 2023; Ong & Ram, 2017). This theory predicts that when mean affect levels are more desirable (i.e., high mean positive affect, low mean negative affect) greater variability is associated with worse health and as mean affect levels become less desirable (i.e., low mean positive affect, high mean negative affect), the greater variability to worse health association is reduced^1^ (Fig. 1). This moderation may take place because, for individuals with more desirable affect, high levels of affect variability might reflect that the desirable emotions experienced are simply a function of the environment and that affect regulation skills to maintain a consistently high level of desirable affect are lacking (as originally explained by the Fragile Positive Affect Theory (Ong & Ram, 2017) but extended here to apply to both valences). In contrast, individuals with less desirable affect likely already have poor health (Pacella et al., 2018; Sirois & Burg, 2003; Suls & Bunde, 2005; Willroth et al., 2020), and thus increasing levels of variability may have little to no added impact on health.

**Fig. 1:**
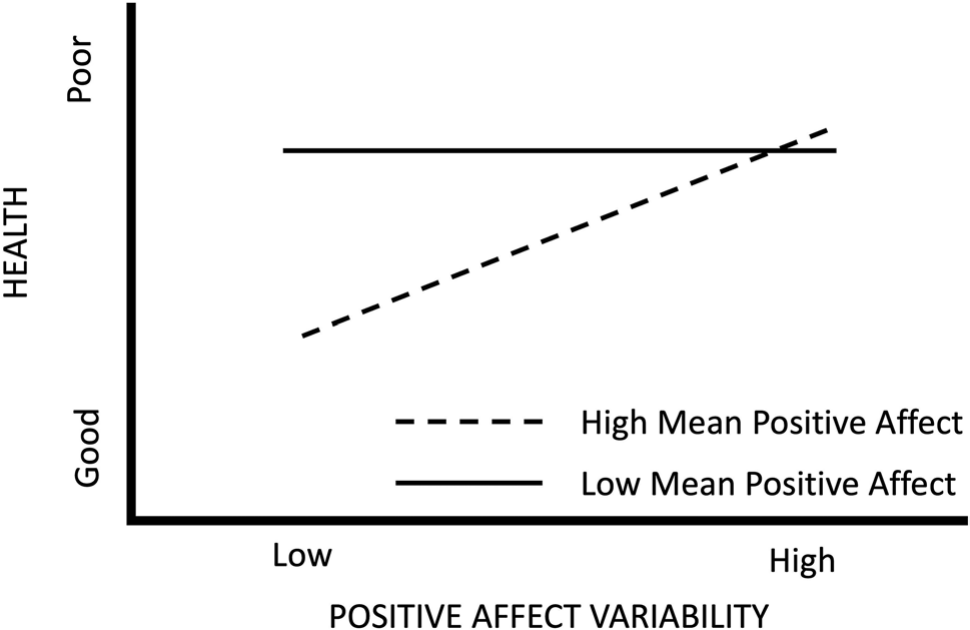
Example of predictions made under the Fragile Desirable Affect Theory

Indeed, a small but growing body of work has shown that mean affect moderates the association between affect variability and physical health/health-relevant outcomes (Jenkins et al., 2018, 2023; Jones et al., 2020). These investigations suggest that it is primarily in the context of high mean positive affect or low mean negative affect that as variability increases, the likelihood of adverse effects increases (e.g., lower antibody response, higher inflammation, more chronic conditions, more medications, worse self-rated physical health). In the mental health literature, two studies have tested this mean by variability interaction, finding that at more desirable mean affect levels (lower negative affect/higher positive affect), more variability is associated with greater depressive symptoms (Maciejewski et al., 2022; Maher et al., 2018). Therefore, in the current investigation, we examined the moderating role of mean affect on the association between affect variability and several mental health outcomes.

However, questions remain as to whether variability reflects adaptive responsiveness to the environment. Thus, some investigations about physiology have shown that it is primarily the extreme highs or lows of variability that are associated with poor outcomes (e.g., less desirable cortisol profiles (Human et al., 2015), greater inflammation (Jones et al., 2020)) while moderate levels of variability are associated with better health. Too much variability may reflect poor emotion regulation, while too little variability may reflect a lack of responsiveness to environmental demands. Thus, we test the quadratic effects of affect variability in addition to the linear ones, on mental health given that quadratic effects have yet to be applied to mental health.

Finally, most affect variability and mental health studies examine cross-sectional associations, which cannot test whether variability predicts long-term outcomes. The few exceptions to the cross-sectional work demonstrate that negative affect variability is associated with depressive symptoms in follow-up periods over the course of more than a year (Wichers et al., 2010) and greater positive and negative affect variability are associated with greater depressive symptoms at follow up periods spanning 6 months to 5 years (Maciejewski et al., 2022). However, previous work from the first and second waves of the Midlife in the United States (MIDUS) Study reported that although greater variability in positive and negative affect was concurrently associated with worse psychological distress, only negative affect variability was associated with long-term psychological distress up to 10 years later (Hardy & Segerstrom, 2017). Thus, there are consistent associations between negative affect variability and mental health across time, but conflicting longitudinal evidence for positive affect variability.

Building on Hardy and Segerstrom (2017) with a larger and more racially diverse sample, the present investigation used cross-sectional and longitudinal data across 10 years from the second and third waves of the MIDUS Study to examine how day-to-day affect variability predicts mental health. We had the following aims: (1) Following the Stability Theory of Affect (Gruber et al., 2013; Hardy & Segerstrom, 2017; Houben et al., 2015), we hypothesized greater variability in positive and negative affect would be associated with poorer self-rated mental health and a higher likelihood of depression, panic disorder, and mental health professional visits in linear models. (2) Extending this work, and drawing from the Fragile Desirable Affect Theory (Jenkins et al., 2023; Ong & Ram, 2017), we tested if linear relationships between variability and worse mental health are moderated by mean affect. We predicted variability-mental health associations would primarily take place at more desirable mean affect levels (i.e., higher positive, lower negative affect). (3) Given initial evidence linking quadratic variability to physical health (Human et al., 2015; Jones et al., 2020), we tested curvilinear variability associations with mental health, and whether mean affect moderates these non-linear links.

## Method

### Participants

Participants included individuals from the second wave (MIDUS II, including a subsample of African Americans from Milwaukee; 2004–2006 (Ryff et al., 2007, 2008)) and third wave (MIDUS III; 2013–2014 (Ryff et al., 2015, 2018)) of the MIDUS Study, a national longitudinal study of US adults (data can be accessed at https://www.icpsr.umich.edu/web/pages/). At MIDUS II, a subsample of respondents participated in the National Study of Daily Experiences (NSDE II [2004–2009]; *n* = 2,022), where they reported on their daily experiences for 8 consecutive days. For the present investigation, of the 2,022 original NSDE II participants, 44 were excluded from analyses due to having fewer than 4 days of affect data (as needed for computing affect mean and variability) or missing sociodemographic data (*n* = 302). The longitudinal analyses further excluded participants who did not participate in MIDUS III (*n* = 255) or had missing sociodemographic data (*n* = 150). The final sample for the cross-sectional analyses contained 1,676 participants, while the longitudinal analyses included 1,271 participants. The average time between participation in the NSDE II and MIDUS III was 7.3 years (*SD* = 1.3).

### Measures

#### Affect

Affect was assessed by 13 positive (e.g., cheerful, attentive) and 14 negative (e.g., nervous, upset) items selected from validated measures (Mroczek & Kolarz, 1998) and based on theory (Watson et al., 1988). Participants were asked on a scale from 0 (*none of the time*) to 4 (*all of the time*) how much of the time they felt each emotion that day. Affect measures were completed once per day for 8 consecutive days. Following prior work, half of the affect data (i.e., 4 of 8 time points) was required to compute affect mean and variability levels (Jenkins et al., 2023; Klaiber et al., 2021).

##### Affect Mean and Variability

Positive affect was first calculated for each day by averaging the scores on the 13 positive affect items from the corresponding day. Next, the average across all available days was taken to create the overall mean positive affect value used in analyses. The same process was used to calculate a mean negative affect value using the 14 negative affect items. The affect scales in the NSDE have acceptable between-person reliability (*R*_kF_ = .99 for positive affect, .97 for negative affect) and within-person reliability (*R*_C_ = .86 for positive affect, .77 for negative affect; Scott et al., 2020).

Next, the standard deviation of the positive affect and negative affect scores for the available days was calculated to form positive affect and negative affect variability, respectively. Standard deviation was calculated using all the available data points for each participant using Formula 1. This resulted in a metric that could be interpreted as “average distance from the mean,” with higher values reflecting greater average distances (i.e., greater affect variability) and lower values reflecting smaller average distances (i.e., less affect variability). The standard deviation was selected because it is commonly used as an indicator of affect variability and is easy to interpret (Eid & Diener, 1999). Moreover, research suggests that other metrics of affect variability, which are typically more complicated, result in the same interpretations as research using the standard deviation (Dejonckheere et al., 2019). Further, the standard deviation is very tightly correlated with other affect variability metrics. For example, the correlation between the standard deviation and the root mean square of successive differences (another common affect variability metric) is approximately *r* = .90 (Jenkins et al., 2018).

$$\textrm{Formula}\ 1:\mathit{SD}=\sqrt{\frac{\sum_{\textrm{i}=1}^{\textrm{n}}{\left({x}_{\textrm{i}}-\overline{x}\right)}^2}{n\hbox{--} 1}}$$

#### Mental Health Outcomes

Mental health outcomes were self-reported in MIDUS II and III. Outcomes that included diagnoses (specifically, depression and panic disorder) were based on scales from Wang et al. (2000), which followed DSM-III criteria.^2^

##### Depression

As done in previous studies (Kessler et al., 2004; Kessler et al., 1999), the depression scale was comprised of depressed affect and anhedonia. To assess depressed affect, participants were first asked, “During the past 12 months, was there ever a time when you felt sad, blue, or depressed for two weeks or more in a row?” Participants who responded “yes” were asked how long the feelings usually lasted, how often they felt that way, and if they experienced a list of seven symptoms. If participants responded they felt sad, blue, or depressed “all day long” or “most of the day,” felt this way “every day” or “almost every day,” and reported “yes” for at least four of the seven depressed affect symptoms, they were categorized as having depressed affect.

Regarding anhedonia, participants were first asked, “During the past 12 months, was there ever a time lasting two weeks or more when you lost interest in most things like hobbies, work, or activities that usually give you pleasure?” Participants who responded “yes” were asked how long they felt that way, how often they felt that way, and if they experienced a list of six symptoms. If participants responded that they felt a loss of interest in most things “all day long” or “most of the day,” felt this way “everyday” or “almost every day,” and reported “yes” for at least four of the six symptoms, they were categorized as having anhedonia.

The final depression outcome was a binary variable: participants were coded as “1” if they were categorized as having depressed affect and/or anhedonia and coded as “0” otherwise. In MIDUS II, 9.1% (*n* = 153) of the total sample was classified as having depression compared to 8.7% (*n* = 110) in MIDUS III.

##### Panic Disorder

As done in previous studies (Wang et al., 2000), panic disorder was assessed by first inquiring whether, in the past 12 months, participants had a panic attack because they felt frightened or for no reason, and when they were not in danger nor the center of attention. If so, they were asked whether they experienced each of six different symptoms (e.g., your heart pounds) during the attacks. Participants were coded as “1” if they reported experiencing three or more symptoms during the attacks and “0” otherwise. In MIDUS II, 6.3% (*n* = 105) of the total sample was classified as having panic disorder, compared to 5.7% (*n* = 72) of the sample at MIDUS III.

##### Self-Rated Mental Health

Participants indicated the state of their mental health with a single item. Possible responses were “poor,” “fair,” “good,” “very good,” or “excellent.” In MIDUS II, most of the sample reported “excellent” (27.7%; *n* = 465), “very good” (37.6%; *n* = 631), or “good” (26.8%; *n* = 449) mental health. In total, 6.9% (*n* = 115) reported their mental health as “fair” and 1% as “poor” (*n* = 16).

##### Saw a Mental Health Professional

Participants were asked how many times they had seen a mental health professional (e.g., psychiatrist, psychologist, professional counselor, marriage therapist, social worker) about their emotional or mental health in the past 12 months.^3^ Most participants (92% at both MIDUS II and MIDUS III) did not see a mental health professional. Participants were coded as “1” if they saw a mental health professional at least once in the past 12 months and “0” otherwise.

### Covariates

Sociodemographic covariates included age, sex, race, education, income, and marital status. Participants denoted their main racial origins as one of six categories: White (reference group), Black/African American, Native American or Alaska Native Aleutian Islander/Eskimo, Asian, Native Hawaiian or Pacific Islander, or other. Education was collapsed into six categories: less than 9th grade (reference group), some high school, high school graduate/GED, some college/AA degree, bachelor’s degree, or higher than bachelor’s degree. Household income at MIDUS II was reported in dollars and collapsed into six categories following the US Census Bureau’s breakdown: <$20,000 (reference group), $20,000–$44,999, $45,000–$139,999, $140,000–$149,999, $150,000–$199,999, or $200,000 and above. Marital status was reported as married (reference group), separated, divorced, widowed, or never married. The cross-sectional analyses controlled for the covariates reported in MIDUS II, whereas the longitudinal analyses controlled for the covariates reported in MIDUS III. The longitudinal models predicting MIDUS III outcomes additionally controlled for the respective health outcome at MIDUS II (e.g., models predicting depression at MIDUS III controlled for depression at MIDUS II).

### Statistical Analyses

All statistical analyses were performed in R Version 4.0.2 (R Core Team, 2022). Means, standard deviations, *t*-tests, and Pearson correlations were used to calculate descriptive statistics of the affect measures. For the primary analyses, mean affect and affect variability were *z*-scored so that regression coefficients would reflect standardized beta values, so that effects could be comparable across predictors, to reduce the negative influences of skewed predictors, and to help decrease the impact of extreme values. Logistic regression was used to model the 0–1 binary outcomes of depression, panic disorder, and mental health professional visits; odds ratios were used for effect sizes. Linear regression modeled self-rated mental health. Significant interaction terms were probed using regions of significance tests with the Johnson-Neyman technique (Rast et al., 2014). This technique indicates at exactly which values of the moderator the association between the independent and dependent variables is significant, as well as the direction of that association. We report these results using percentiles and only do so for values that are within our range of data (despite the Johnson-Neyman reporting values outside the data range). For graphing interactions, we graph the lines demonstrating the independent (*z*-scored affect variability) and dependent (mental health) variable associations at the 10th, 30th, 50th, 70th, and 90th percentiles of the moderator (*z*-scored mean affect) variable. For mean positive affect, the percentiles represent scale points at 1.77, 2.45, 2.79, 3.08, and 3.61 for concurrent analyses and 1.78, 2.45, 2.77, 3.03, and 3.56 for longitudinal analyses. For mean negative affect, the percentiles represent scale points at 0.02, 0.06, 0.12, 0.22, and 0.47 for concurrent analyses and 0.03, 0.07, 0.13, 0.21, and 0.43 for longitudinal analyses. Additionally, for logistic regression models, regions of significance tests were analyzed on a linear scale (e.g., log-odds) but graphed with curvilinear probabilities for ease of interpretation. Significant quadratic associations were graphed to visually inspect the curved association between the independent and dependent variables. Power analyses revealed that a sample size above 1,000 would be sufficient to achieve at least 80% power to detect small-medium effect sizes at the alpha .05 level (Cohen, 1988).

Four separate models were used for each outcome to test the linear effect of affect variability adjusting for mean affect (Model 1), the interaction between mean level and linear affect variability (Model 2), the quadratic effect of affect variability adjusting for mean affect (Model 3), and the interaction between mean level and quadratic affect variability (Model 4) for each valence. These four models were conducted for positive and negative affect for each cross-sectional outcome and then for positive and negative affect for each longitudinal outcome. All models controlled for the covariates described above. Finally, sensitivity analyses tested whether the effects held when controlling for the number of daily diary assessments and time differences between NSDE II and MIDUS III. The pattern of effects remained the same, so only the results from the main analyses are presented.

## Results

### Descriptive Statistics

About half of the sample (56.6%) was female. Participants’ self-identified racial origins were 84.6% White, 11.6% Black and/or African American, 1.3% Native American or Alaska Native Aleutian Islander/Eskimo, 0.3% Asian, and 2.3% Native Hawaiian/Pacific Islander or other (inclusive of multiracial). Most participants were married (69.3%) and had educational attainment higher than a high school diploma (70.6%). At MIDUS II, ages ranged from 33 to 83 years (*M* = 55; *SD* = 12). The longitudinal sample had similar demographic characteristics, with the exception of age (*M =* 63; *SD* = 11).

**Fig. 2:**
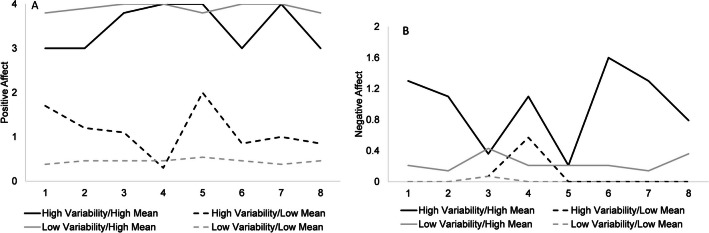
For positive affect (**A**), we graphed at the 85th percentile for high values and the 15th percentile for low values. Given that mean negative affect and negative affect variability were so highly correlated, we had to then graph at the 65th percentile for high values and the 25th percentile for low values to capture participants with the high-low combinations (**B**)

In the cross-sectional sample, mean positive affect was significantly higher than mean negative affect (*t* = 137.07, *p* < .001, 95% confidence interval [CI] of the difference [2.49, 2.56], Cohen’s *d* = 4.80; Table 1). Similarly, positive affect variability was higher than negative affect variability (*t* = 26.94, *p* < .001, 95% CI of the difference [0.15, 0.18], Cohen’s *d* = 0.93). Given that we would be examining the interaction effects between affect mean and variability, we graphed four participants with different combinations of high vs. low mean and variability for each affective valence (Fig. 2A and B). Further, individuals higher in mean positive affect tended to be lower in mean negative affect and have lower affect variability, regardless of valence (Table 1). In contrast, those higher in mean negative affect had higher affect variability irrespective of valence. Participants with greater positive affect variability also had higher levels of negative affect variability. The longitudinal sample’s affect measures showed the same patterns.

**Table 1:** Descriptive statistics of mean affect and variability measures

Measure	Mean	*SD*	2	3	4
1. Mean positive affect	2.73	0.70	−0.56 (−0.58, −0.52)	−0.23 (−0.28, −0.18)	−0.46 (−0.49, −0.42)
2. Mean negative affect	0.19	0.25		0.24 (0.19, 0.28)	0.75 (0.73, 0.77)
3. Positive affect variability	0.34	0.20			0.40 (0.36, 0.44)
4. Negative affect variability	0.17	0.15			

The last three columns depict the correlation matrix of the variables. 95% confidence intervals are in parentheses

### Concurrent Mental Health Outcomes

#### Linear Affect Variability

Greater positive affect variability was associated with worse mental health across the four outcomes (Table 2 Model 1), whereas greater negative affect variability was associated only with an increased probability of having panic disorder (OR = 1.26, 95% CI [1.00, 1.58], *p* = .046; Table 3 Model 1). Participants with greater positive affect variability were more likely to have depression (OR *=* 1.29, 95% CI [1.09, 1.51], *p* = .002), were more likely to have panic disorder (OR = 1.40, 95% CI [1.17, 1.68], *p* < .001 ), had worse self-rated mental health (*β* = 0.07, 95% CI [0.02, 0.11], *p* = .002), and were more likely to have seen a mental health professional in the past 12 months (*OR =* 1.25, 95% CI [1.05, 1.48], *p* = .012).

**Table 2:** Positive affect tests of the Stability Theory of Affect and the Fragile Desirable Affect Theory for concurrent mental health

Outcome	Mean	Variability	Mean × Variability	Variability × Variability	Mean × Variability × Variability
Depression					
Model 1	0.551*	1.287*			
Model 2	0.553*	1.272*	0.980		
Model 3	0.553*	1.328*		0.983	
Model 4	0.552*	1.312*	0.965	0.983	1.006
Panic disorder					
Model 1	0.741*	1.401*			
Model 2	0.721*	1.472*	1.111		
Model 3	0.741*	1.403*		0.959	
Model 4	0.771*	1.620*	1.270	0.936	0.919
Self-rated mental health					
Model 1	−0.352*	0.067*			
Model 2	−0.351*	0.067*	0.003		
Model 3	−0.348*	0.062*		−0.011	
Model 4	−0.348*	0.080*	−0.003	−0.012	−0.002
Saw a mental health professional					
Model 1	0.613*	1.250*			
Model 2	0.593*	1.461*	1.326*		
Model 3	0.964*	1.023*		0.995	
Model 4	0.961*	1.024*	1.008	0.998	1.003

Standardized regression estimates are presented for self-rated mental health. Odds ratios are presented for all other outcomes. All models controlled for sociodemographic covariates at MIDUS II. Please see Supplemental Table S1 for models without covariates

*p < .05

**Table 3:** Negative affect tests of the Stability Theory of Affect and the Fragile Desirable Affect Theory for concurrent mental health

Outcome	Mean	Variability	Mean* Variability	Variability × Variability	Mean × Variability × Variability
Depression					
Model 1	1.649*	1.118			
Model 2	1.953*	1.264*	0.875*		
Model 3	1.593*	1.385*		0.931	
Model 4	2.295*	1.241	0.714*	0.985	1.042
Panic disorder					
Model 1	1.263*	1.260*			
Model 2	1.649*	1.528*	0.824*		
Model 3	1.195	2.077*		0.850*	
Model 4	1.490*	1.878*	0.835	0.899	1.018
Self-rated mental health					
Model 1	0.303*	0.016			
Model 2	0.364*	0.036	−0.047*		
Model 3	0.296*	0.088*		−0.033*	
Model 4	0.359*	0.050	−0.060	−0.017	0.007
Saw a mental health professional					
Model 1	1.824*	0.873			
Model 2	2.017*	0.955	0.924		
Model 3	1.086*	0.980		0.996	
Model 4	1.074*	0.987	1.009	0.993	0.999

Standardized regression estimates are presented for self-rated mental health. Odds ratios are presented for all other outcomes. All models controlled for sociodemographic covariates at MIDUS II. Please see Supplemental Table S2 for models without covariates

*p < .05

#### Mean Affect Moderating Linear Affect Variability

**Fig. 3:**
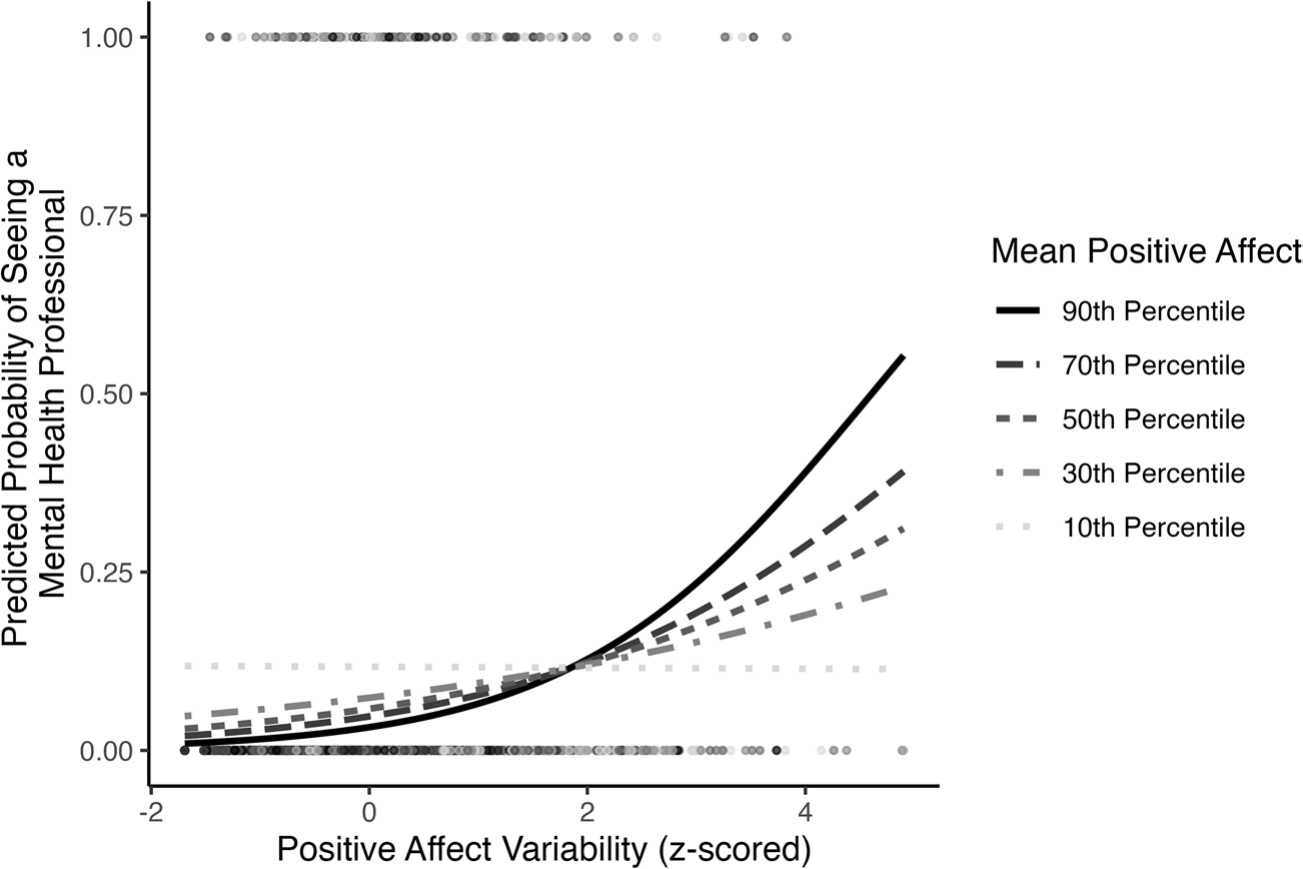
Positive affect mean by variability interaction predicting concurrent probability of seeing a mental health professional

Mean positive affect moderated the association between positive affect variability and seeing a mental health professional (OR = 1.33, 95% CI [1.11, 1.59], *p* = .002; Table 2 Model 2; Fig. 3). Regions of significance tests demonstrated that the slope between positive affect variability and seeing a mental health professional was positive when mean positive affect was above the 21st percentile (Supplemental Fig. S1). When mean positive affect was extremely low (below the 0.5th percentile), the relationship changed to negative.

**Fig. 4:**
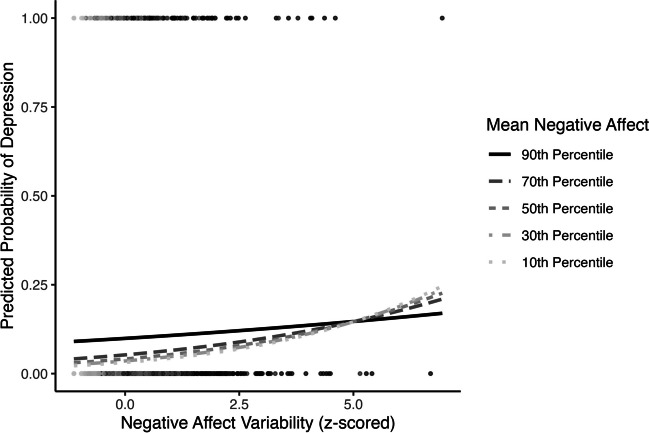
Negative affect mean by variability interaction predicting concurrent probability of depression

Mean negative affect moderated the association between negative affect variability and three of the four health outcomes. First, mean negative affect moderated the association between negative affect variability and depression (OR = 0.88, 95% CI [0.79, 0.96], *p* = .004; Table 3 Model 2; Fig. 4). Regions of significance tests demonstrated that the slope between negative affect variability and depression was positive when mean negative affect was below the 70th percentile (Supplemental Fig. S2). When mean negative affect was above the 99th percentile, the relationship changed to negative.

**Fig. 5:**
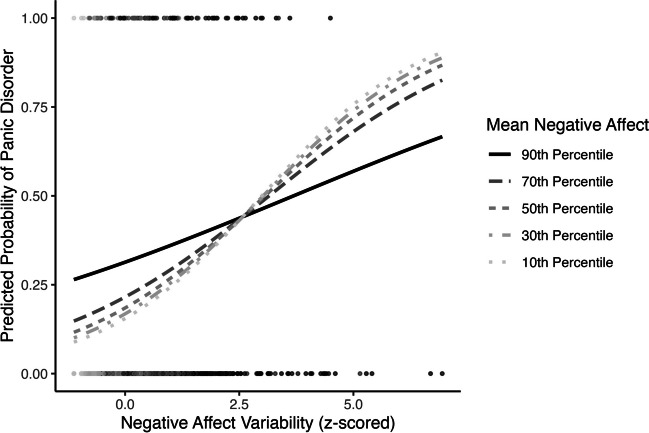
Negative affect mean by variability interaction predicting concurrent probability of panic disorder

Second, mean negative affect significantly moderated the association between negative affect variability and panic disorder (OR = 0.82, 95% CI [0.72, 0.92], *p* = .002; Table 3 Model 2; see Fig. 5). Regions of significance tests showed that the slope between negative affect variability and panic disorder was positive when mean negative affect was below the 89th percentile (Supplemental Fig. S3). At extremely high values of mean negative affect (above the 99th percentile), the slope between negative affect variability and panic disorder became negative.

**Fig. 6:**
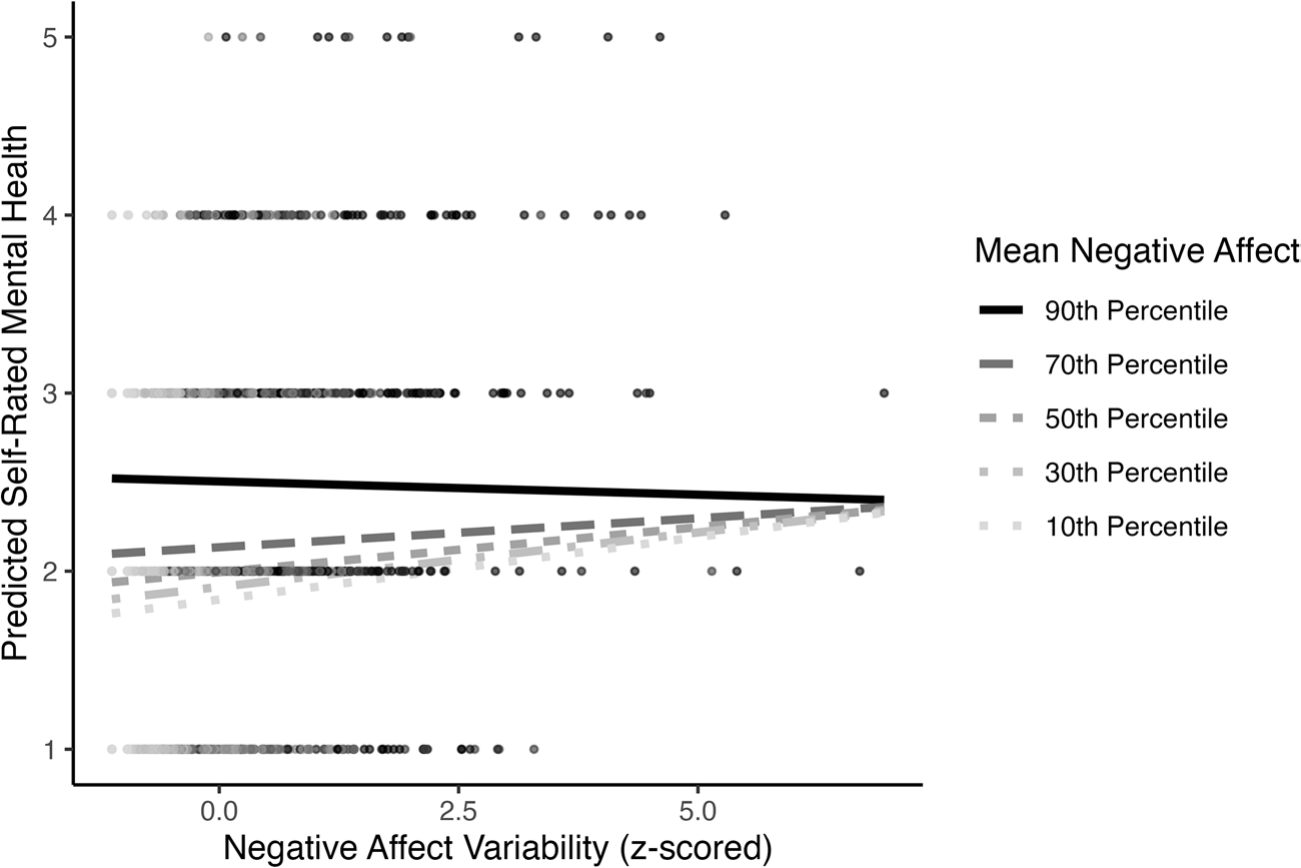
Negative affect mean by variability interaction predicting concurrent self-rated mental health

Third, mean negative affect significantly moderated the association between negative affect variability and self-rated mental health (*β* = −0.05, 95% CI [−0.07, −0.02], *p* = < .001; Table 3 Model 2; Fig. 6). The slope between negative affect variability and self-rated mental health was positive only at low values (below the 10th percentile) and became negative at extremely high values (above the 97th percentile) of mean negative affect (Supplemental Fig. S4).

#### Quadratic Affect Variability

**Fig. 7:**
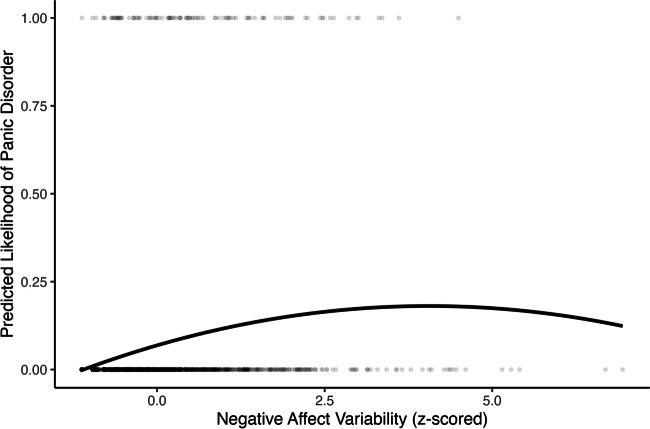
Quadratic association between negative affect variability and concurrent probability of panic disorder

**Fig. 8:**
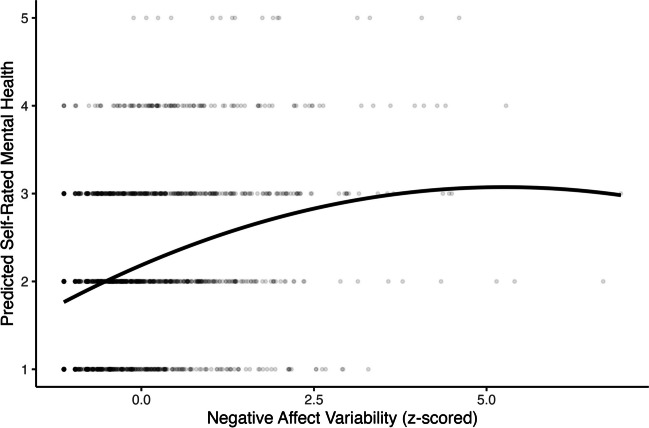
Quadratic association between negative affect variability and concurrent self-rated mental health

Negative affect variability had a negative quadratic association with panic disorder (OR *=* 0.85, 95% CI [0.75, 0.93], *p* = .003) and self-rated mental health (*β =* −0.03, 95% CI [−0.05, −0.01], *p* = .001; Table 3 Model 3; Figs. 7 and 8). Negative affect variability was associated with a higher probability of panic disorder and worse self-rated mental health when variability scores were below approximately the fourth and fifth standard deviation of observed scores, respectively. Above the fourth and fifth standard deviation, negative affect variability was associated with a lower probability of panic disorder and better self-rated mental health, respectively. No quadratic positive affect variability nor other quadrative negative affect variability predictors were significantly associated with our outcomes, *p*s > .05 (Tables 2 and 3 Model 3).

#### Mean Affect Moderating Quadratic Affect Variability

Mean affect did not moderate any of the associations between the quadratic terms of affect variability and our mental health outcomes, *p*s > .05 (Tables 2 and 3 Model 4).

### Long-Term Mental Health Outcomes

#### Linear Affect Variability

In models controlling for the respective health outcome at MIDUS II, positive affect variability significantly predicted three of the four mental health outcomes (Table 4), whereas negative affect variability significantly predicted two of the four outcomes (Table 5). Participants with greater positive and/or negative affect variability were more likely to have subsequent depression (positive affect variability OR = 1.42, 95% CI [1.14, 1.75], *p* = .002; negative affect variability OR = 1.45, 95% CI [1.07, 1.95], *p* = .015) and worse self-rated mental health (positive affect variability *β* = 0.10, 95% CI [0.04, 0.16], *p* < .001; negative affect variability *β* = 0.10, *SE* = 0.04, *p* = .028; Tables 4 and 5 Model 1). Additionally, participants with greater positive affect variability were more likely to have panic disorder (OR = 1.45, 95% CI [1.12, 1.87], *p* = .004; Table 4 Model 1).

**Table 4:** Positive affect tests of the Stability Theory of Affect and the Fragile Desirable Affect Theory for long-term mental health

Outcome	Mean	Variability	Mean × Variability	Variability × Variability	Mean × Variability × Variability
Depression					
Model 1	0.660*	1.415*			
Model 2	0.636*	1.697	1.364*		
Model 3	0.661*	1.454*		0.984	
Model 4	0.671*	1.681*	1.549*	0.981	0.914
Panic disorder					
Model 1	0.584*	1.449*			
Model 2	0.582*	1.470*	1.024		
Model 3	0.648*	1.818*		0.920	
Model 4	0.602*	1.780*	0.941	0.981	1.097
Self-rated mental health					
Model 1	−0.247*	0.101*			
Model 2	−0.245*	0.108*	0.028		
Model 3	−0.246*	0.118*		−0.019	
Model 4	−0.236*	0.113*	0.025	−0.018	−0.011
Saw a mental health professional					
Model 1	0.796	1.045			
Model 2	0.798	1.088	1.090		
Model 3	0.961	0.905		0.984	
Model 4	0.966	0.893	1.151	1.019	0.991

Standardized regression estimates are presented for self-rated mental health. Odds ratios are presented for all other outcomes. All models controlled for sociodemographic covariates at MIDUS III and respective health outcome at MIDUS II. Please see Supplemental Table S3 for models without covariates

*p < 0.05

**Table 5:** Negative affect tests of the Stability Theory of Affect and the Fragile Desirable Affect Theory for long-term mental health

Outcome	Mean	Variability	Mean × Variability	Variability × Variability	Mean × Variability × Variability
Depression					
Model 1	1.229	1.450*			
Model 2	1.285	1.478*	0.962		
Model 3	1.220	1.551*		0.968	
Model 4	1.382	1.474	0.827	0.978	0.410
Panic disorder					
Model 1	1.461*	1.204			
Model 2	1.875*	1.365	0.806		
Model 3	1.309	2.547*		0.732*	
Model 4	1.717	2.190*	0.751	0.812	1.038
Self-rated mental health					
Model 1	0.171*	0.098*			
Model 2	0.241*	0.106*	−0.072*		
Model 3	0.167*	0.148*		−0.038	
Model 4	0.309*	0.037	−0.175*	0.035	0.021
Saw a mental health professional					
Model 1	1.057	1.397			
Model 2	1.256	1.438*	0.943		
Model 3	1.162	0.868		1.013	
Model 4	1.226	0.847	1.089	1.087	0.947

Standardized regression estimates are presented for self-rated mental health. Odds ratios are presented for all other outcomes. All models controlled for sociodemographic covariates at MIDUS III and respective health outcome at MIDUS II. Please see Supplemental Table S4 for models without covariates

*p < 0.05

#### Mean Affect Moderating Linear Affect Variability

**Fig. 9:**
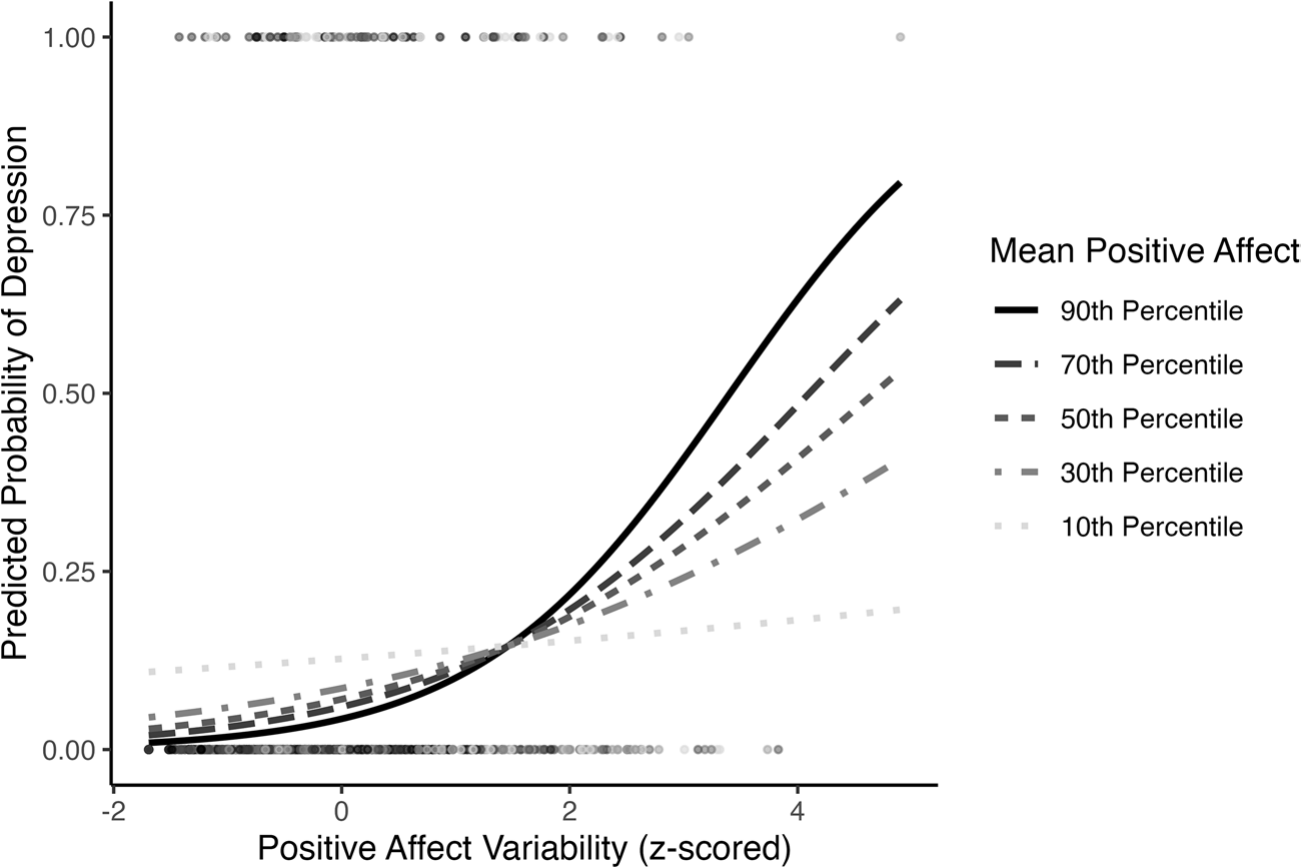
Positive affect mean by variability interaction predicting long-term probability of depression

In models controlling for the respective health outcome at MIDUS II, mean positive affect moderated the association between positive affect variability and the probability of depression (OR = 1.36, 95% CI [1.08, 1.73], *p* = .009; Table 4 Model 2; Fig. 9). Regions of significance tests demonstrated that the slope between positive affect variability and the probability of depression was positive and significant when mean positive affect was greater than the 15th percentile (Supplemental Fig. S5).

**Fig. 10:**
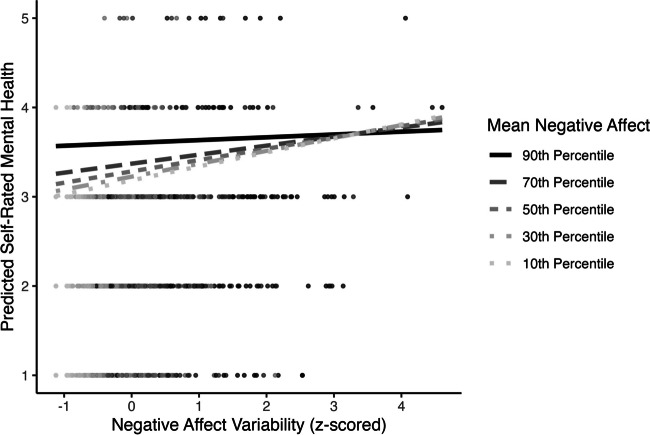
Negative affect mean by variability interaction predicting long-term self-rated mental health

Additionally, in models controlling for the respective health outcome at MIDUS II, mean negative affect moderated the association between negative affect variability and self-rated mental health (*β* = −0.07, 95% CI [−0.12, −0.02], *p* = .004; Table 5 Model 2; Fig. 10). Regions of significance tests demonstrated that the slope between negative affect variability and self-rated mental health was positive and significant when mean negative affect was below the 78th percentile (Supplemental Fig. S6).

#### Quadratic Affect Variability

**Fig. 11:**
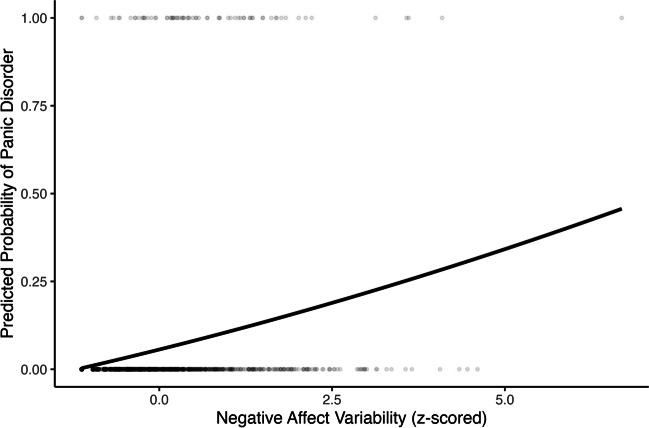
Quadratic association between negative affect variability and long-term probability of panic disorder

In models controlling for the respective health outcome at MIDUS II, there was a significant negative quadratic association between negative affect variability and long-term probability of panic disorder (OR = 0.73, 95% CI [0.59, 0.88], *p* = .002; Table 5 Model 3; Fig. 11). Generally, greater negative affect variability was associated with a greater likelihood of panic disorder while the curvature of the quadratic association was only minorly concave up. No quadratic positive affect variability nor other quadrative negative affect variability predictors were significantly associated with the outcomes, *p*s > .05 (Tables 4 and 5 Model 3).

#### Mean Affect Moderating Quadratic Affect Variability

In models controlling for the respective health outcome at MIDUS II, mean affect did not moderate associations between the quadratic terms of affect variability and the mental health outcomes, *p*s > .05 (Tables 4 and 5 Model 4).

## Discussion

In line with previous work (Gruber et al., 2013; Houben et al., 2015), we found that positive affect variability was associated concurrently with all four mental health outcomes. Greater positive affect variability was associated with a higher likelihood of depression, panic disorder, and seeing a mental health professional, as well as worse self-rated mental health. However, negative affect variability was only concurrently associated with a higher likelihood of having a panic disorder and was not associated with the other three outcomes. Individuals tend to experience much more variability in their positive affect states as compared to negative affect, which may account for the larger number of main effects seen for positive affect variability here and in other work (e.g., Gruber et al., 2013; Human et al., 2015; Jenkins et al., 2018). Findings from this study also demonstrate that affect variability predicts mental health outcomes up to 10 years later, even when adjusting for baseline mental health. However, the effects were different from those seen with concurrent associations. Specifically, positive affect variability continued to be associated with a higher probability of depression and panic disorder, as well as worse self-rated mental health but no longer preceded seeing a mental health professional. Greater negative affect variability predicted worse self-rated health and a higher likelihood of depression but no longer predicted the likelihood of a panic disorder.

Consistent with the Fragile Desirable Affect Theory, there were interactions between affect variability and mean levels. Mean negative affect moderated the association between negative affect variability and three of the four concurrent outcomes (depression, panic disorder, and self-rated mental health) and one of the four long-term outcomes (self-rated mental health). Mean positive affect moderated the association between positive affect variability and concurrent likelihood of seeing a mental health professional and long-term depression. Generally, for individuals with less desirable affect (high mean negative or low mean positive), there was no relationship between variability and mental health. However, for those with more desirable affect (low mean negative or high mean positive), greater variability associated with poorer health. Specifically, as shown using the Johnson-Neyman technique (Rast et al., 2014), the bottom 70–89% on mean negative affect and the top 79–85% on mean positive affect exhibited this linkage between higher variability and worse mental health. Albeit, for concurrent self-rated mental health, it was only the bottom 10% on mean negative affect that showed the variability-worse health association. Although there were instances in which greater affect variability was associated with better mental health, this only occurred for people at the extremeness on less desirable affect (i.e., top 3% or less for mean negative affect, bottom 0.5% or less for mean positive affect).

The statistical relationship between mean levels and variability may have implications for how we interpret our interaction results. Specifically, the correlation between positive affect mean and variability (*r* = −.23) was weaker than that for negative affect (*r* = .75). While the correlation itself does not impact the likelihood of an interaction, it may influence the distribution of values on the two interacting variables. Examination of value combinations revealed an even distribution across levels of positive affect mean and variability (Supplemental Table S5). This indicates that, despite the negative correlation, some participants still scored high on both variables while others scored low on both. However, the strong positive correlation between negative affect mean and variability meant that most participants scored either high on both variables or low on both (Supplemental Table S6). For example, no participants scored in the highest variability and lowest mean negative affect quintiles (and vice versa), likely due to floor effects limiting variability when mean levels are very low. Consequently, the simple slopes for positive affect are relevant across all independent variable values since participants cover all mean-variability combinations. But for negative affect, the simple slope at low mean levels represents those with low variability, while the simple slope at high mean levels represents those with high variability. Parsing these nuanced patterns assists in the interpretation of the moderation effects and underscores the differential implications of the relationships between variability and mean affect by valence.

Prior research has demonstrated curvilinear associations between affect variability and health-relevant outcomes, with very high or low variability conferring risk (Human et al., 2015; Jones et al., 2020). However, our analyses found limited evidence for such quadratic relationships. Only three quadratic effects emerged for negative affect variability. Further inspection of these effects showed that greater negative affect variability was largely associated with worse mental health, aligning with the observed linear relationships. It was only at extremes exceeding four or five standard deviations above the mean that greater negative variability predicted better concurrent health. Similarly, long-term associations followed the typical positive linear pattern, with the curve of the line only modestly concave. No quadratic effects were found for positive affect variability. Additionally, mean affect levels did not moderate any curvilinear associations between variability and mental health. These results indicate overall linear effects of greater affect variability on poorer mental health, with minimal impact of curvilinear patterns. Still, future research should continue investigating the shape of curvilinear relationships to determine their significance. Careful modeling of the effects at very high or low levels of variability will help establish boundaries and provide a more nuanced understanding of risk patterns.

Future investigations can also build on the current study by considering other metrics of variability and/or collecting data over the course of more days and/or more time points throughout the day to provide a more stable and fine-grained assessment of affect variability. Future research should also include evaluations of affect regulation skills and environmental contexts to better distinguish between adaptive (Bonanno & Burton, 2013; Kashdan & Rottenberg, 2010) and maladaptive (Koval, Ogrinz, Kuppens, Van Den Bergh, Tuerlinckx, & Sütterlin, 2013; Vannucci et al., 2019) affect variability. Further, while we controlled for mental health at MIDUS II when predicting MIDUS III mental health outcomes, reverse causality cannot be ruled.^4^

This investigation had several notable strengths, including the use of a large, diverse national sample with substantial African American representation. Additionally, examining mean affect as a moderator allowed for a more nuanced understanding of how variability links to mental health across different affect levels. Another strength was the breadth of analyses across various mental health outcomes and evaluating variability separately by valence, aligning with established practices in this research domain (e.g., Gruber et al., 2013; Jenkins et al., 2018, 2020; Koval, Pe, Meers, & Kuppens, 2013; Peeters et al., 2006).

Finally, identifying longitudinal relationships is an initial but crucial step in elucidating the mechanisms linking variability to mental health over time. While not tested here, likely mediators include health behaviors (e.g., Leger et al., 2019; Maher et al., 2018; Mohr et al., 2015; Wen et al., 2018), biological processes (e.g., Human et al., 2015; Jenkins et al., 2018; Koval, Ogrinz, Kuppens, Van Den Bergh, Tuerlinckx, & Sütterlin, 2013), and social interactions (e.g., Miller & Pilkonis, 2006; Urganci et al., 2022). Examining variability’s long-term mental health associations may be informative given these mediators require time to unfold. Moving forward, further examination of longitudinal links and temporal mechanisms is warranted, in addition to experimental tests of whether regulating variability improves mental health for those with more favorable mean affect. The current results provide the groundwork for advancing research on variability and affect regulation in the context of mental health.

### Supplementary Information

ESM 1 (DOCX 678 kb)

## Footnotes

1 And, in some cases reversed (e.g., Jenkins et al., 2023; Maher et al., 2018). Although reasons for this effect are less understood.

2 While generalized anxiety disorder (GAD) was assessed in our participant sample, the rate of GAD and even GAD symptoms were very low (MIDUS II: 34 participants (2%) qualified as having GAD and 45 participants (2.7%) reported 1 or more symptoms; MIDUS III: 21 participants (1.7%) qualified as having GAD and 31 participants (2.4%) reported 1 or more symptoms).

3 Mental health service use in other sectors (not just mental health professionals) was assessed in our sample. Thus, for the interested reader, we report the results of mental health service use more broadly in our supplemental material.

4 Although please see our reverse direction analyses in our supplemental material for further discussion on this.

## Additional Information

### Data Availability

Data can be accessed at https://www.icpsr.umich.edu/web/pages/.
