# Supplementary material for: Mean Affect Moderates the Association between Affect Variability and Mental Health
Source: Affect Sci. 2024 Jun 13;5(2):99–114. doi: 10.1007/s42761-024-00238-0 (PMC11264645; doi:10.1007/s42761-024-00238-0)
Supplement: Supplementary file 1 — (DOCX 678 kb) [file 42761_2024_238_MOESM1_ESM.docx]

**Supplemental Material Table of Contents**

Mental Health Service Use Across Five Sectors 2

Models without Covariates 3

Johnson-Neyman Results Figures 7

Mean by Variability Spread of Data 13

Reverse Direction Analyses 14

**Mental Health Service Use Across Five Sectors**

Here, we report results when examining participants’ mental health service use that is not limited to only seeing mental health professionals. Participants were asked how many times they sought mental health services from five different sectors in the past 12 months, including a psychiatrist, general doctor, counselor (inclusive of psychologist, marriage therapist, or social worker), religious advisor, or self-help group. Participants were coded as “1” if they endorsed using at least one of the five mental health sectors and “0” otherwise. Most participants (MIDUS II: 71%; MIDUS III: 81%) did not seek any mental health service. Among those who did, the majority reported going to two sectors.

Concurrently, participants with greater positive affect variability were more likely to have sought mental health services in the past 12 months (*OR =* 1.14, 95% CI [1.02, 1.27], *p* = .021). There were no other linear or curvilinear associations between affect variability and seeking mental health services, nor were there interactions between mean affect and linear affect variability, *p*s > 0.05.

**Models without Covariates**

**Table S1.** Positive Affect Tests of the Stability Theory of Affect and the Fragile Desirable Affect Theory for Concurrent Mental Health.

| Outcome | Mean | Variability | Mean*Variability | Variability*Variability | Mean*Variability*Variability |
| --- | --- | --- | --- | --- | --- |
| Depression |  |  |  |  |  |
| Model 1 | 0.55 * | 1.33 * |  |  |  |
| Model 2 | 0.55 * | 1.30 * | 0.96 |  |  |
| Model 3 | 0.55 * | 1.45 * |  | 0.96 |  |
| Model 4 | 0.55 * | 1.41 * | 0.93 | 0.96 | 1.01 |
| Panic Disorder |  |  |  |  |  |
| Model 1 | 0.71 * | 1.46 * |  |  |  |
| Model 2 | 0.69 * | 1.53 * | 1.09 |  |  |
| Model 3 | 0.71 * | 1.69 * |  | 0.93 |  |
| Model 4 | 0.73 * | 1.78 * | 1.20 | 0.91 | 0.93 |
| Self-Rated Mental Health |  |  |  |  |  |
| Model 1 | -0.34 * | 0.11 * |  |  |  |
| Model 2 | -0.34 * | 0.11 * | -0.02 |  |  |
| Model 3 | -0.34 * | 0.11 * |  | 0.00 |  |
| Model 4 | -0.34 * | 0.11 * | -0.02 | 0.00 | 0.00 |
| Saw a Mental Health Professional |  |  |  |  |  |
| Model 1 | 0.57 * | 1.25 * |  |  |  |
| Model 2 | 0.55 * | 1.45 * | 1.29 * |  |  |
| Model 3 | 0.58 * | 1.50 * |  | 0.91 |  |
| Model 4 | 0.57 * | 1.74 * | 1.36 * | 0.90 | 0.96 |

*Note.* Standardized regression estimates are presented for self-rated mental health. Odds ratios are presented for all other outcomes. **p* < 0.05.

**Table S2.** Negative Affect Tests of the Stability Theory of Affect and the Fragile Desirable Affect Theory for Concurrent Mental Health.

| Outcome | Mean | Variability | Mean*Variability | Variability*Variability | Mean*Variability*Variability |
| --- | --- | --- | --- | --- | --- |
| Depression |  |  |  |  |  |
| Model 1 | 1.52 * | 1.19 |  |  |  |
| Model 2 | 1.88 * | 1.40 * | 0.86 * |  |  |
| Model 3 | 1.47 * | 1.55 * |  | 0.92 * |  |
| Model 4 | 2.21 * | 1.36 | 0.70 * | 0.99 | 1.04 * |
| Panic Disorder |  |  |  |  |  |
| Model 1 | 1.28 * | 1.35 * |  |  |  |
| Model 2 | 1.69 * | 1.64 * | 0.82 * |  |  |
| Model 3 | 1.22 * | 2.15 * |  | 0.86 * |  |
| Model 4 | 1.64 * | 1.85 * | 0.80 | 0.94 | 1.02 |
| Self-Rated Mental Health |  |  |  |  |  |
| Model 1 | 0.31 * | 0.04 |  |  |  |
| Model 2 | 0.36 * | 0.05 | -0.04 * |  |  |
| Model 3 | 0.31 * | 0.10 * |  | -0.03 * |  |
| Model 4 | 0.35 * | 0.07 | -0.04 | -0.02 | 0.00 |
| Saw a Mental Health Professional |  |  |  |  |  |
| Model 1 | 1.86 * | 0.92 |  |  |  |
| Model 2 | 2.09 * | 1.03 | 0.91 * |  |  |
| Model 3 | 1.80 * | 1.19 |  | 0.91 |  |
| Model 4 | 2.02 * | 1.18 | 0.87 | 0.91 | 1.03 |

*Note.* Standardized regression estimates are presented for self-rated mental health. Odds ratios are presented for all other outcomes. **p* < 0.05.

**Table S3.** Positive Affect Tests of the Stability Theory of Affect and the Fragile Desirable Affect Theory for Long-Term Mental Health.

| Outcome | Mean | Variability | Mean*Variability | Variability*Variability | Mean*Variability*Variability |
| --- | --- | --- | --- | --- | --- |
| Depression |  |  |  |  |  |
| Model 1 | 0.57 * | 1.45 * |  |  |  |
| Model 2 | 0.54 * | 1.65 * | 1.22 * |  |  |
| Model 3 | 0.57 * | 1.52 * |  | 0.97 |  |
| Model 4 | 0.59 * | 1.82 * | 1.55 * | 0.92 | 0.87 * |
| Panic Disorder |  |  |  |  |  |
| Model 1 | 0.60 * | 1.75 * |  |  |  |
| Model 2 | 0.59 * | 1.85 * | 1.09 |  |  |
| Model 3 | 0.61 * | 1.88 * |  | 0.97 |  |
| Model 4 | 0.58 * | 1.88 * | 1.01 | 1.00 | 1.04 |
| Self-Rated Mental Health |  |  |  |  |  |
| Model 1 | -0.29 * | 0.14 * |  |  |  |
| Model 2 | -0.29 * | 0.14 * | -0.03 |  |  |
| Model 3 | -0.29 * | 0.15 * |  | -0.01 |  |
| Model 4 | -0.29 * | 0.14 * | -0.04 | -0.02 | -0.01 |
| Saw a Mental Health Professional |  |  |  |  |  |
| Model 1 | 0.61 * | 1.19 |  |  |  |
| Model 2 | 0.60 * | 1.32 * | 1.21 |  |  |
| Model 3 | 0.61 * | 1.26 |  | 0.96 |  |
| Model 4 | 0.63 * | 1.38 * | 1.34 | 0.95 | 0.93 |

*Note.* Standardized regression estimates are presented for self-rated mental health. Odds ratios are presented for all other outcomes. **p* < 0.05.

**Table S4.** Negative Affect Tests of the Stability Theory of Affect and the Fragile Desirable Affect Theory for Long-Term Mental Health.

| Outcome | Mean | Variability | Mean*Variability | Variability*Variability | Mean*Variability*Variability |
| --- | --- | --- | --- | --- | --- |
| Depression |  |  |  |  |  |
| Model 1 | 1.52 * | 1.29 * |  |  |  |
| Model 2 | 1.70 * | 1.41 * | 0.91 |  |  |
| Model 3 | 1.49 * | 1.58 * |  | 0.93 |  |
| Model 4 | 1.61 * | 1.52 * | 0.93 | 0.95 | 1.01 |
| Panic Disorder |  |  |  |  |  |
| Model 1 | 1.34 * | 1.53 * |  |  |  |
| Model 2 | 1.60 * | 1.75 * | 0.87 * |  |  |
| Model 3 | 1.30 * | 2.03 * |  | 0.91 |  |
| Model 4 | 1.80 * | 2.32 * | 0.66 * | 0.83 | 1.09 * |
| Self-Rated Mental Health |  |  |  |  |  |
| Model 1 | 0.26 * | 0.11 * |  |  |  |
| Model 2 | 0.28 * | 0.12 * | -0.02 |  |  |
| Model 3 | 0.25 * | 0.12 * |  | -0.01 |  |
| Model 4 | 0.33 * | 0.08 | -0.09 | 0.02 | 0.01 |
| Saw a Mental Health Professional |  |  |  |  |  |
| Model 1 | 1.54 * | 1.22 |  |  |  |
| Model 2 | 1.67 * | 1.3 | 0.93 |  |  |
| Model 3 | 1.50 * | 1.61 * |  | 0.90 |  |
| Model 4 | 1.21 | 1.88 * | 1.18 | 0.80 | 1.00 |

*Note.* Standardized regression estimates are presented for self-rated mental health. Odds ratios are presented for all other outcomes. **p* < 0.05.

**Johnson-Neyman Results Figures**

**Figure S1.** Johnson-Neyman interval for positive affect mean by variability interaction predicting concurrent probability of seeing a mental health professional.


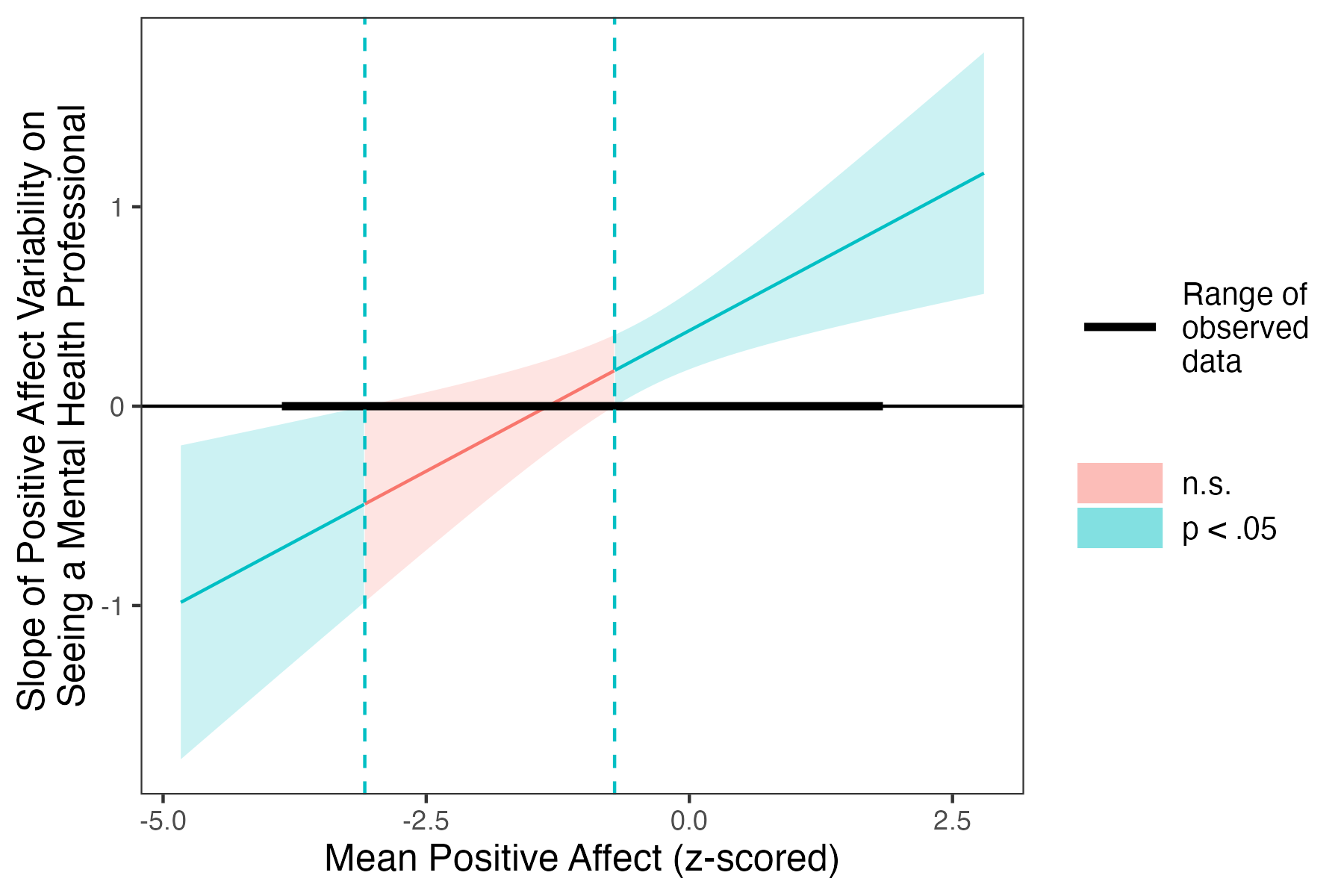


*Note.* The slope between positive affect variability and seeing a mental health professional was positive when mean positive affect was above the 21^st^ percentile. When mean positive affect was extremely low (below the 0.5^th^ percentile), the relationship changed to negative.

**Figure S2.** Johnson-Neyman interval for negative affect mean by variability interaction predicting concurrent probability of depression.


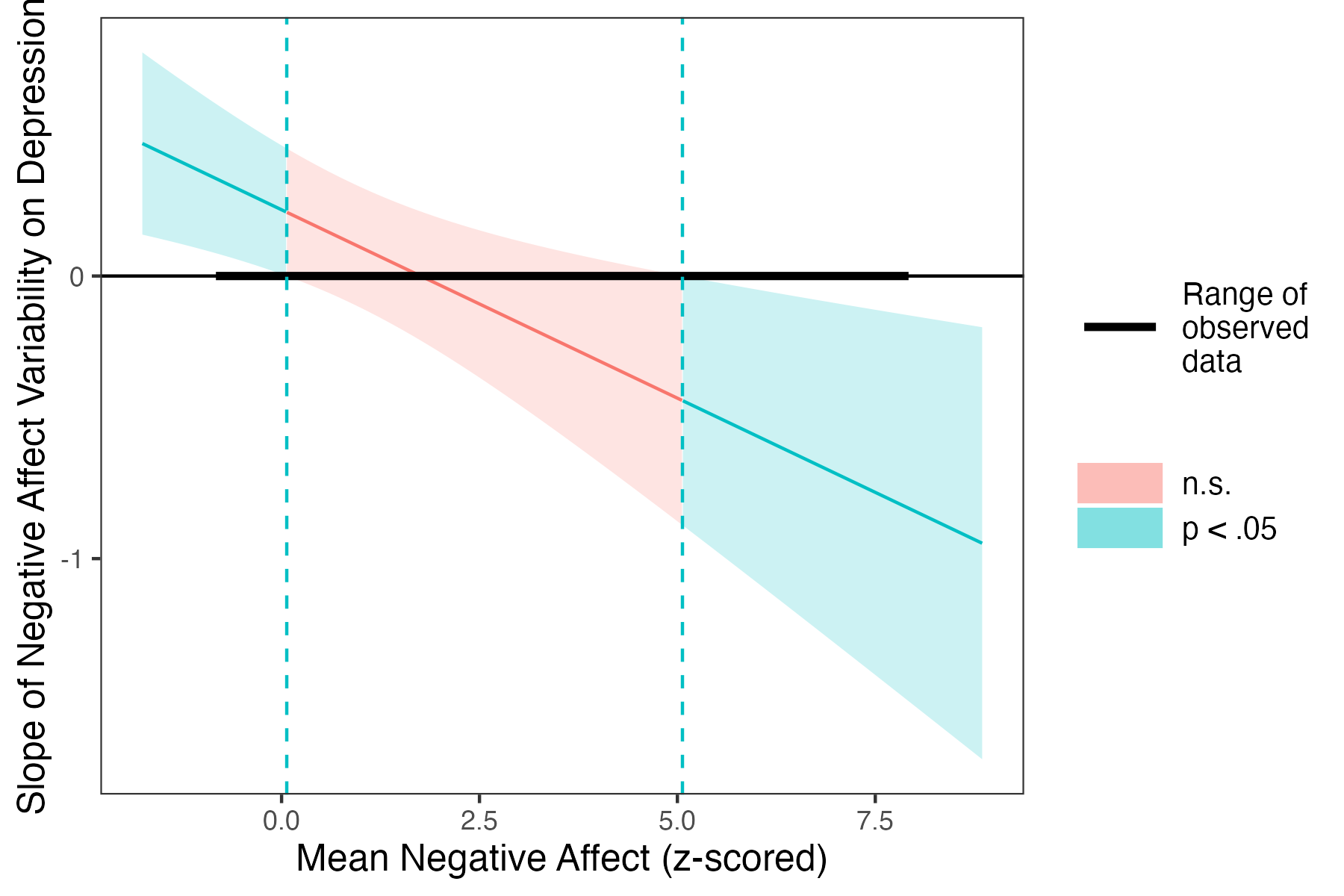


*Note.* The slope between negative affect variability and depression was positive when mean negative affect was below the 70th percentile. When mean negative affect was above the 99th percentile, the relationship changed to negative.

**Figure S3.** Johnson-Neyman interval for negative affect mean by variability interaction predicting concurrent probability of panic disorder.


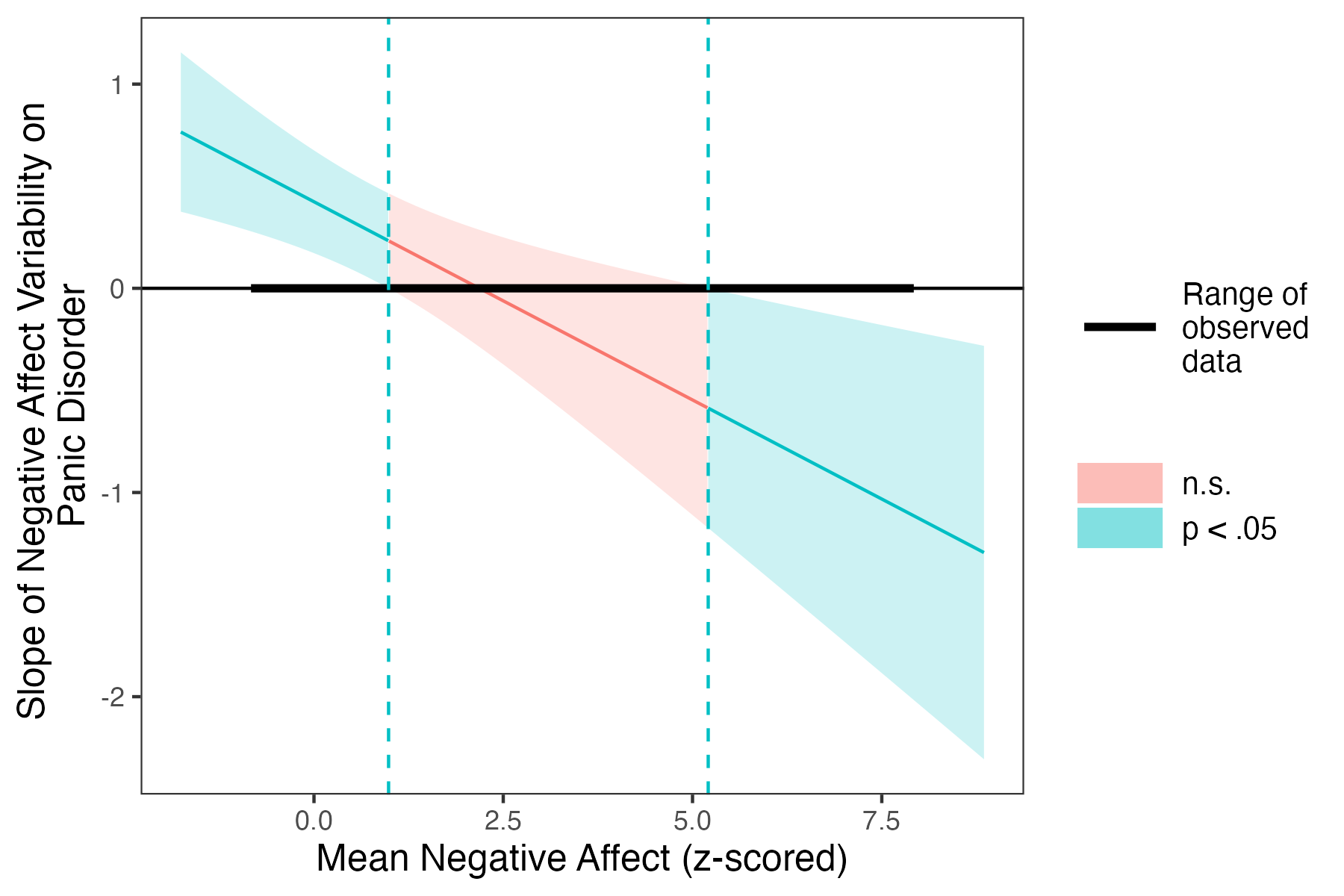


*Note.* The slope between negative affect variability and panic disorder was positive when mean negative affect was below the 89^th^ percentile. At extremely high values of mean negative affect (above the 99^th^ percentile), the slope between negative affect variability and panic disorder became negative.

**Figure S4.** Johnson-Neyman interval for negative affect mean by variability interaction predicting concurrent self-rated mental health.


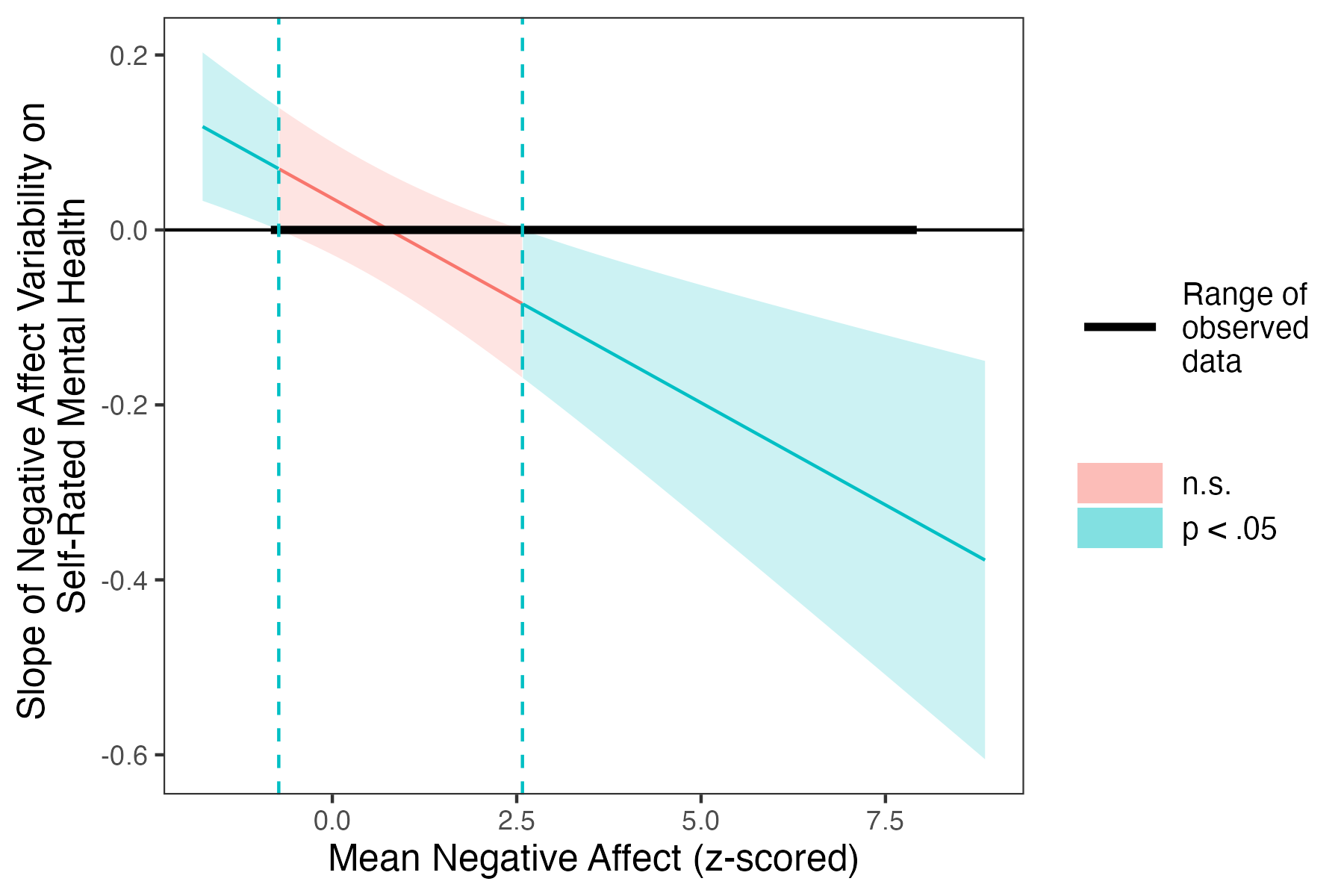


*Note.* The slope between negative affect variability and self-rated mental health was positive only at low values (below the 10^th^ percentile) and became negative at extremely high values (above the 97^th^ percentile) of mean negative affect.

**Figure S5.** Johnson-Neyman interval for positive affect mean by variability interaction predicting long-term probability of depression.


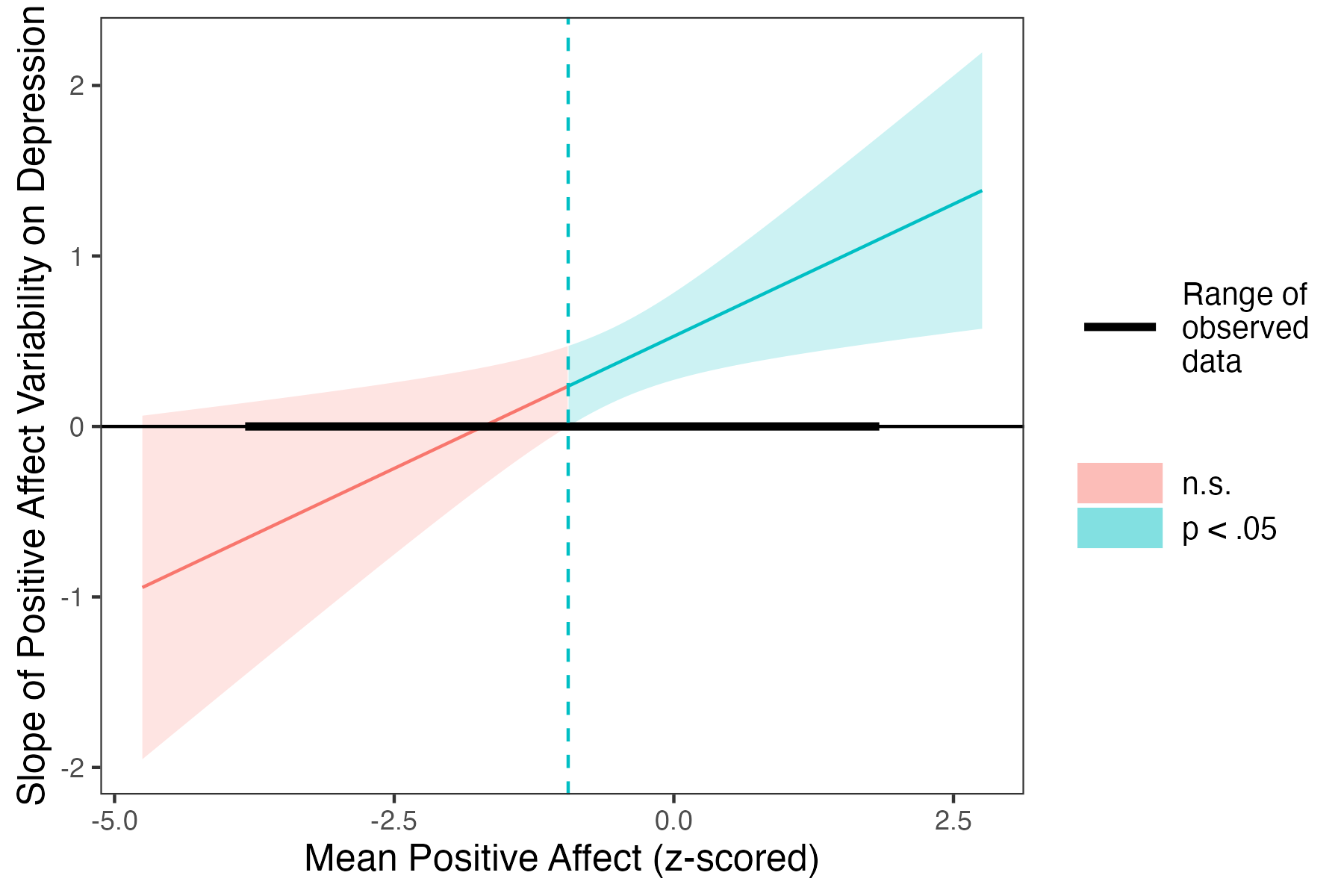


*Note.* The slope between positive affect variability and the probability of depression was positive and significant when mean positive affect was greater than the 15^th^ percentile.

**Figure S6.** Johnson-Neyman interval for negative affect mean by variability interaction predicting long-term self-rated mental health.


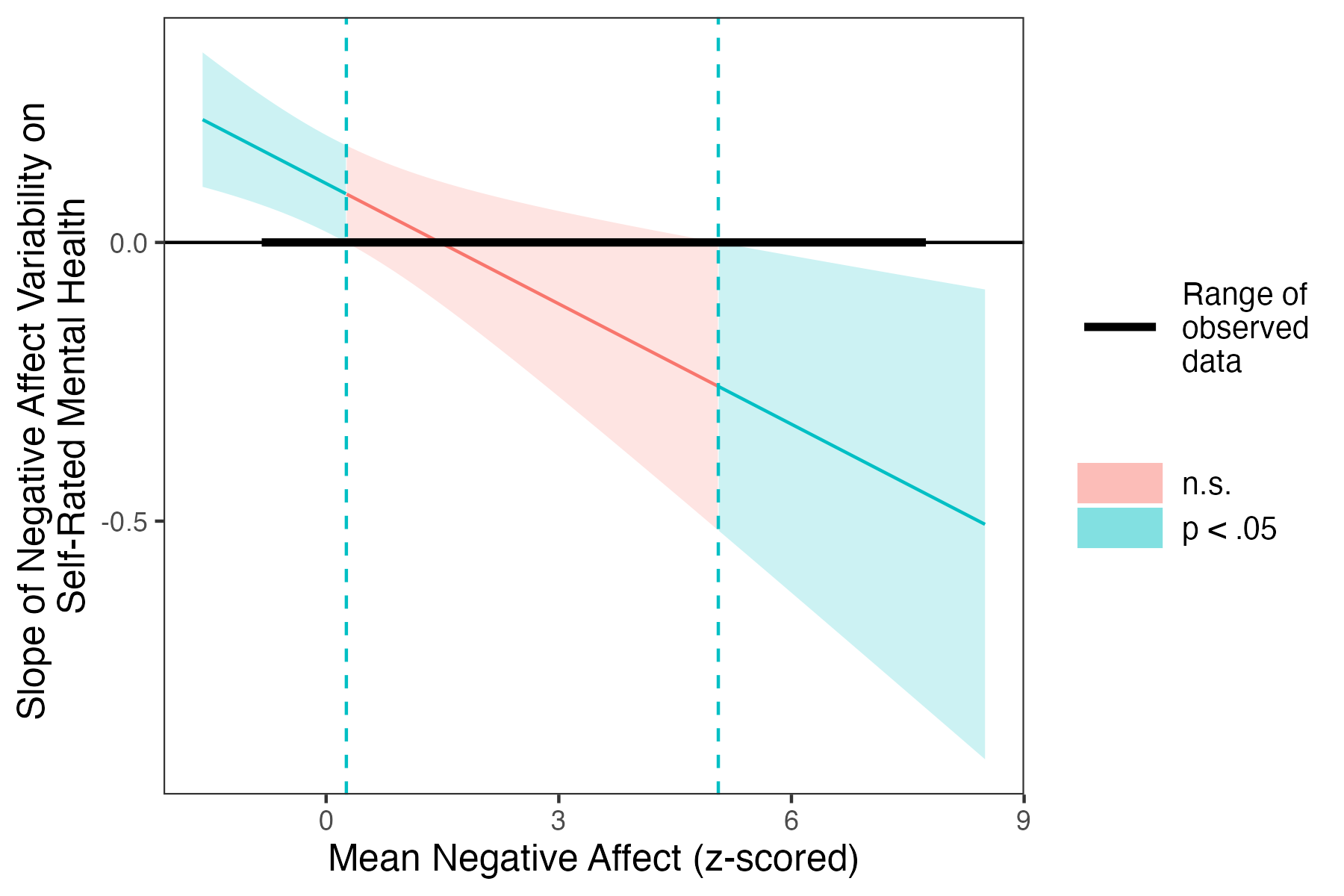


*Note.* The slope between negative affect variability and self-rated mental health was positive and significant when mean negative affect was below the 78^th^ percentile.

**Mean by Variability Spread of Data**

**Table S5.** Table depicting sample size and percentage of sample in each quintile pairing between variability and mean level for positive affect.

|  |  | Positive Affect Variability Quintile | | | | |
| --- | --- | --- | --- | --- | --- | --- |
|  |  | **0-19%** | **20-39%** | **40-59%** | **60-79%** | **80-99%** |
| Mean Positive Affect Quintile | **0-19%** | 20 (1%) | 53 (3%) | 75 (4%) | 88 (5%) | 103(6%) |
|  | **20-39%** | 51 (3%) | 69 (4%) | 58 (3%) | 69 (4%) | 85 (5%) |
|  | **40-59%** | 102 (6%) | 73 (4%) | 56 (3%) | 56 (3%) | 52 (3%) |
|  | **60-79%** | 81 (5%) | 73 (4%) | 65 (4%) | 57 (3%) | 56 (3%) |
|  | **80-99%** | 82 (5%) | 67 (4%) | 81 (5%) | 65 (4%) | 39 (2%) |

**Table S6.** Table depicting sample size and percentage of sample in each quintile pairing between variability and mean level for negative affect.

|  |  | Negative Affect Variability Quintile | | | | |
| --- | --- | --- | --- | --- | --- | --- |
|  |  | **0-19%** | **20-39%** | **40-59%** | **60-79%** | **80-99%** |
| Mean Negative Affect Quintile | **0-19%** | 295 (18%) | 53 (3%) | 0 (0%) | 0 (0%) | 0 (0%) |
|  | **20-39%** | 32 (2%) | 191 (11%) | 88 (5%) | 12 (1%) | 0 (0%) |
|  | **40-59%** | 7 (0.4%) | 66 (4%) | 142 (8%) | 106 (6%) | 15 (1%) |
|  | **60-79%** | 3 (0.2%) | 17 (1%) | 78 (5%) | 145 (9%) | 91 (5%) |
|  | **80-99%** | 0 (0%) | 7 (0.4%) | 27 (2%) | 72 (4%) | 229 (14%) |

**Reverse Direction Analyses**

We conducted a supplemental analysis using mental health at MIDUS II (2004-2006) to predict affect variability at NSDE III (2017-2019) while controlling for NSDE II affect variability. We found that seeing a mental health professional and worse self-rated mental health at MIDUS II were each associated with greater negative affect variability at NSDE III. Worse self-rated mental health and panic disorder at MIDUS II were each also associated with greater positive affect variability at NSDE III (Table S7). Although long-term associations between affect variability and mental health are similar to those presented in the paper for self-rated mental health and panic disorder, they are not the same for depression and mental health service use, giving credibility to the idea that it’s not just simply reverse causality. However, there are likely bidirectional relationships between mental health and affect variability. The purpose of the current paper was to investigate one of these directions (i.e., whether affect variability is related to subsequent mental health), although future research could continue to explore the bidirectional nature of these relationships.

**Table S7.** Mental health at MIDUS II predicting positive and negative affect variability at NSDE III.

| Variables | Depression | Panic Disorder | Self-Rated Mental Health | Saw a Mental Health Professional |
| --- | --- | --- | --- | --- |
| NA Variability | 0.01 |  |  |  |
| PA Variability | 0.04 |  |  |  |
| NA Variability |  | 0.02 |  |  |
| PA Variability |  | 0.09 * |  |  |
| NA Variability |  |  | 0.01 * |  |
| PA Variability |  |  | 0.01 * |  |
| NA Variability |  |  |  | 0.05 * |
| PA Variability |  |  |  | 0.03 |

*Note.* Unstandardized regression estimates presented. All models controlled for sociodemographic covariates at MIDUS II. **p* < 0.05.
